# Response: Commentary on the effects of hypoxia on energy substrate use during exercise

**DOI:** 10.1186/s12970-019-0330-7

**Published:** 2019-12-19

**Authors:** Alex Griffiths, Oliver Shannon, Jamie Matu, Roderick King, Kevin Deighton, John P. O’Hara

**Affiliations:** 1 Research Institute for Sport, Physical Activity and Leisure, Leeds Beckett University, Leeds, LS6 3QS UK; 2 Human Nutrition Research Centre, Institute of Cellular Medicine, Newcastle University, Leech Building, Framlington Place, Newcastle Upon Tyne, NE2 4HH UK; 3 School of Clinical and Applied Science, Leeds Beckett University, Leeds, LS1 3HE UK

**Keywords:** Altitude, Carbohydrate, Fat, Oxidation, Relative, Absolute, RER

## Abstract

**Background:**

A recent commentary has been published on our meta-analysis, which investigated substrate oxidation during exercise matched for *relative* intensities in hypoxia compared with normoxia. Within this commentary, the authors proposed that exercise matched for *absolute* intensities in hypoxia compared with normoxia, should have been included within the analysis, as this model provides a more suitable experimental design when considering nutritional interventions in hypoxia.

**Main body:**

Within this response, we provide a rationale for the use of exercise matched for *relative* intensities in hypoxia compared with normoxia. Specifically, we argue that this model provides a physiological stimulus replicable of real world situations, by reducing the absolute workload undertaken in hypoxia. Further, the use of exercise matched for *relative* intensities isolates the metabolic response to hypoxia, rather than the increased relative exercise intensity experienced in hypoxia when utilising exercise matched for *absolute* intensities. In addition, we also report previously unpublished data analysed at the time of the original meta-analysis, assessing substrate oxidation during exercise matched for *absolute* intensities in hypoxia compared with normoxia.

**Conclusion:**

An increased reliance on carbohydrate oxidation was observed during exercise matched for *absolute* intensities in hypoxia compared with normoxia. These data now provide a comparable dataset for the use of researchers and practitioners alike in the design of nutritional interventions for relevant populations.

## Background

The authors welcome the constructive feedback provided by Young et al. [1] regarding our recent meta-analysis [2]. Their critique relates to the validity of practical/nutritional applications for relevant populations when informed by substrate oxidation responses during exercise matched for *relative* intensities in hypoxia and normoxia (i.e. exercise is conducted at the same percentage of altitude-specific $\dot{V}$O_2max_). Young et al. [1] suggest that as any given workload in hypoxia requires the same *absolute* energy requirements as normoxia, nutritional strategies for relevant populations should be informed by substrate oxidation responses during exercise matched for *absolute* intensities in hypoxia and normoxia (i.e. exercise is conducted at the same absolute workload in hypoxia and normoxia). However, as the *relative* percentage of $\dot{V}$O_2max_ utilised during sub-maximal exercise of the same absolute workload is higher in hypoxia compared with normoxia [3], muscle metabolic perturbations are increased. Specifically, finite metabolic substrates such as muscle glycogen and phosphocreatine are degraded, subsequently elevating the accumulation of fatigue-associated metabolites such as H^+^, inorganic phosphate and adenosine diphosphate [4]. This effect is potentiated in hypoxia compared with normoxia when using exercise matched for *absolute* exercise intensities. For reasons discussed below, it is our view that the use of *absolute* exercise intensities and the associated physiological stimulus do not reflect real world applications, and the use of exercise matched for *relative* intensities under the same metabolic stimulus is more appropriate.

In order to understand the utilisation of each substrate during exercise at high-altitude (and therefore determine nutritional interventions), it is necessary to isolate the effects of hypoxia (as per *relative* intensities), rather than the effect of an increased exercise intensity (as per *absolute* intensities). During high-altitude sojourns, exercise is not performed at increased exercise intensities, as induced by exercise matched for *absolute* intensities. As a result of physiological and psychological factors, high-altitude mountaineers, military personnel and athletes exercise at a reduced *absolute* workload, to compensate for the reduced oxygen availability experienced at high-altitude, thus matching the same *relative* exercise intensity in hypoxia compared with normoxia. Therefore, for ecological validity, we believe nutritional interventions should be informed by exercise matched for *relative*, rather than *absolute* intensities in hypoxia and normoxia.

In order to justify the use of a specific model, it is important to determine the differences in substrate oxidation between exercise matched for *absolute* and *relative* intensities in hypoxia and normoxia. In addition to the important narrative synthesis provided by Young et al. [1], it is necessary to summarise these findings in a systematic and quantitative manner. As such, we will report and discuss previously unpublished data from our meta-analysis regarding substrate oxidation during exercise matched for *absolute* intensities in hypoxia, compared with normoxia.

## Methods

Methodological details (literature search, inclusion criteria, data abstraction, risk of bias, statistical analysis) of the meta-analysis have been reported previously [2]. The sole difference between data reported in the present manuscript and previously published data is the use of exercise matched for *absolute*, rather than *relative* intensities. In brief, included studies were required to measure respiratory exchange ratio (RER) and/or carbohydrate or fat oxidation. These measures were required to be quantified during exercise in both hypoxic and normoxic environments. Normoxic trials were required to provide a viable within-subjects control (i.e. equivalent measure(s) quantified in the same participants). In order to maintain a comparable dataset to previously published data [2], the search dates for the present manuscript were not updated. *Albeit*, the recent papers by Young et al. [5] and O’Hara et al. [6] were included in the discussion of these data.

## Results

A total of 1743 studies published in peer review journals were identified through database screening as part of the full meta-analysis (*relative* and *absolute* intensities). Following the screening process, a total of 6 studies utilising exercise matched for *absolute* intensities in hypoxia and normoxia were identified as suitable for the meta-analyses. A total of 23 comparisons were made for exercise matched for *absolute* intensities (RER = 7, absolute carbohydrate oxidation = 6, absolute fat oxidation = 4, relative carbohydrate oxidation = 3, relative fat oxidation = 3).

Tables 1 and 2 present changes in RER and substrate oxidation rates respectively, in relation to exercise matched for *absolute* intensities.

**Table 1:** Summary of studies investigating the effect of hypoxia on RER during exercise matched for *absolute* intensity

Study	Participants	Study design	Type of hypoxia	Altitude (m)	Duration of hypoxia	RER
Braun et al. [7]	15 (females)	30 min cycling at SL (50% SL VO_2max_) and hypoxia (65% altitude VO_2max_)	TA	4300	10 days	SL: 0.95 ± 0.01 CH: 0.94 ± 0.02
Katz and Sahlin [8]	8 (males)	5 min exercise at SL (49% SL VO_2max_) and altitude (67% altitude VO_2max_)	NH	4500	22 min	SL: 0.96 ± 0.01 AH: 1.10 ± 0.04
Kelly and Basset [9]	7 (males)	60 min exercise at SL (69% SL VO_2max_) and altitude (78% altitude VO_2max_)	NH	2750	180 min	SL: 0.92 ± 0.05 AH: 0.93 ± 0.04
Lundby and Van Hall [10] A	8 (male = 6, female = 2)	60 min cycling at SL (46% SL VO_2max_) and at altitude (54% SL VO_2max_)	NH	4100	70 min	SL: 0.91 ± 0.01 AH: 0.95 ± 0.02
Lundby and Van Hall [10] B	8 (male = 6, female = 2)	60 min cycling at SL (46% SL VO_2max_) and at altitude (59% altitude VO_2max_)	TA	4100	28 days	SL: 0.91 ± 0.01 CH: 0.94 ± 0.01
Péronnet et al. [11]	5 (males)	80 min cycling at SL (54% SL max) and at altitude (77% altitude VO_2max_)	HH	4300	110 min	SL: 0.92 ± 0.02 AH: 0.97 ± 0.01
Van Hall et al. [12]	6 (male = 5, female = 1)	20 min cycling at SL (46% SL VO_2max_) and altitude (82% altitude VO_2max_)	TA	5620	63 days	SL: 0.92 ± 0.02 CH: 0.92 ± 0.01

Values presented as mean ± SD

*HH* hypobaric hypoxia, *NH* normobaric hypoxia, *TA* terrestrial altitude, *SL* sea level, *AH* acute hypoxia, *CH* chronic hypoxia

**Table 2:** Summary of studies investigating the effect of hypoxia on substrate utilisation during exercise matched for *absolute* intensity

Study	Participants	Study design	Type of hypoxia	Altitude (m)	Duration of exposure	Absolute substrate use (g.min^− 1^)		Relative substrate use (%)	
						CHO oxidation	Fat oxidation	CHO oxidation	Fat oxidation
Braun et al. [7]	15 (females)	30 min cycling at SL (50% SL VO_2max_) and hypoxia (65% altitude VO_2max_)	TA	4300	10 days	SL: 1.38 ± 0.08 CH:1.22 ± 0.09	NM	NM	NM
Kelly and Basset [9]	7 (males)	60 min exercise at SL (69% SL VO_2max_) and altitude (78% altitude VO_2max_)	NH	2750	180 min	SL: 2.27 ± 0.57 AH: 2.30 ± 0.50	SL: 0.46 ± 0.18 AH: 0.34 ± 0.21	NM	NM
Lundby and Van Hall [10] A	8 (male = 6, female = 2)	60 min cycling at SL (46% SL VO2max) and at altitude (54% SL VO_2max_)	NH	4100	70 min	SL: 2.00 ± 0.20 AH: 2.50 ± 0.20	SL: 0.30 ± 0.01 AH: 0.20 ± 0.01	SL: 73.90 ± 2.00 AH: 86.20 ± 2.00	SL: 26.10 ± 2.00 AH: 13.80 ± 2.00
Lundby and Van Hall [10] B	8 (male = 6, female = 2)	60 min cycling at SL (46% SL VO2max) and at altitude (59% altitude VO_2max_)	TA	4100	10 days	SL: 2.00 ± 0.20 CH: 2.30 ± 0.10	SL: 0.30 ± 0.01 CH: 0.20 ± 0.01	SL: 73.90 ± 2.00 CH: 82.20 ± 2.20	SL: 26.10 ± 2.00 CH: 17.80 ± 2.20
Péronnet et al. [11]	5 (males)	80 min cycling at SL (54% SL max) and at altitude (77% altitude VO_2max_)	HH	4300	110 min	SL: 2.18 ± 0.11 AH:2.67 ± 0.10	SL: 0.32 ± 0.08 AH: 0.10 ± 0.03	SL: 75.30 ± 5.20 AH: 92.00 ± 2.10	SL: 24.70 ± 5.20 AH: 8.00 ± 2.10
Van Hall et al. [12]	6 (male = 5, female = 1)	20 min cycling at SL (46% SL VO_2max_) and altitude (82% altitude VO_2max_)	TA	5620	63 days	SL: 2.22 ± 0.34 CH: 2.31 ± 0.14	NM	NM	NM

Values presented as mean ± SD

*HH* hypobaric hypoxia, *NH* normobaric hypoxia, *TA* terrestrial altitude, *SL* sea level, *AH* acute hypoxia, *CH* chronic hypoxia, *CHO* carbohydrate, *NM* not measured

### Participant demographics and study characteristics

Of the 57 participants included in the analysis, 37 were male (76.2%) and 20 were female (23.8%). Age was reported in all studies and ranged from 22 to 28 years old (mean = 25 years). BMI was reported in 5 of the 6 studies and ranged from 22.3 to 25.2 kg·m^−2^. $\dot{V}$O_2max_ was reported in all studies and ranged from 2.61 to 4.30 L.min^−1^ (mean = 3.80 L.min^− 1^).

Exercise duration ranged from 5 min to 80 min (mean = 45 min). Participants in normoxic trials performed exercise at intensities ranging from 46 to 69% of normoxic $\dot{V}$O_2max_ (mean = 52% $\dot{V}$O_2max_) and hypoxic trials were performed at 54–82% hypoxic $\dot{V}$O_2max_ (mean = 69% $\dot{V}$O_2max_). The severity of hypoxia quantified in meters ranged from 2750 to 5620 m (mean = 4200 m).

### Mean difference, heterogeneity, sensitivity and moderator analysis for RER

**Fig. 1:**
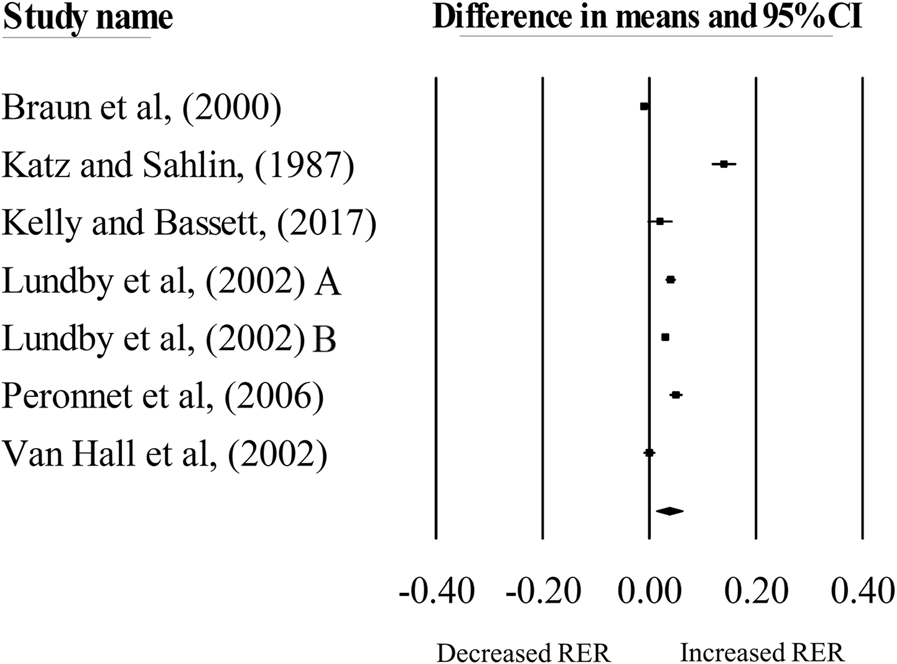
Forest plot of mean differences (means ±95% CI) for studies investigating the effects of hypoxia on RER during exercise matched for absolute intensities. The size of the square represents the relative weight of the trial. CIs are represented by a horizontal line through their representative circles. The diamond quantifies the overall mean difference (means ±95% CI). A and B refer to the different trial arms of each study. Details of which are provided in Table 1

Hypoxic exposure resulted in a significant increase in RER during exercise matched for *absolute* intensities, compared with normoxia (mean difference: 0.04, 95% CI = 0.01 to 0.06; *n* = 7; *p* < 0.01; Fig. 1). The degree of heterogeneity was found to be high between studies (*I*^2^ = 98.57%, Q = 419.47, τ^2^ = 0.001, d_f_ = 6). Sensitivity analysis revealed minor changes only, and these changes did not substantially alter the overall mean effect. Meta-regression analysis revealed that no moderators were significantly associated with RER during exercise matched to *absolute* intensities in hypoxia, compared with normoxia (Additional file 1). Inspection of the funnel plot and Egger’s regression intercept revealed that there was little evidence of small study effects (intercept = 8.70, 95% CI: − 3.10 to 20.50; *p* = 0.12).

### Mean difference, heterogeneity and sensitivity analysis for relative carbohydrate and fat oxidation rates

Hypoxic exposure resulted in a significant increase in relative carbohydrate oxidation during exercise matched for *absolute* intensities, compared with normoxia (mean difference: 12.1, 95% CI: 8.3 to 16.0%; *n* = 3, *p* < 0.01; Additional file 2). Sensitivity analysis revealed minor changes only, and these changes did not substantially alter the overall mean difference. Inspection of the funnel plot and Egger’s regression intercept revealed that there was little evidence of small study effects (intercept = 7.59, 95% CI: − 60.78 to 75.97; *p* = 0.39).

Hypoxic exposure resulted in a significant decrease in relative fat oxidation during exercise matched for *absolute* intensities, compared with normoxia (mean difference: -12.7, 95% CI: − 16.9 to − 8.4%; *n* = 3, *p* < 0.01; Additional file 3). The degree of heterogeneity was found to be high between studies (*I*^2^ = 95.94%, Q = 49.27, τ^2^ = 13.02, d_f_ = 2). Sensitivity analysis revealed minor changes only, and these changes did not substantially alter the overall mean difference. Inspection of the funnel plot and Egger’s regression intercept revealed that there was little evidence of small study effects (intercept = − 8.89, 95% CI: − 72.57 to 54.80; *p* = 0.33).

### Mean difference, heterogeneity and sensitivity analysis for absolute carbohydrate and fat oxidation rates

**Fig. 2:**
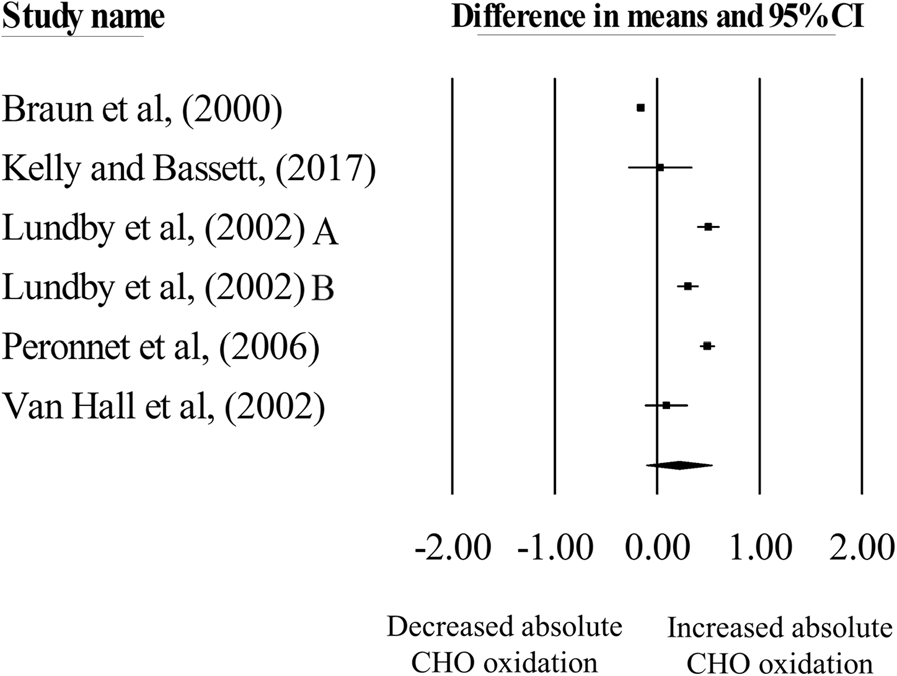
Forest plot of mean differences (means ±95% CI) for studies investigating the effects of hypoxia on absolute carbohydrate oxidation during exercise matched for absolute intensities. The size of the square represents the relative weight of the trial. CIs are represented by a horizontal line through their representative circles. The diamond quantifies the overall mean difference (means ±95% CI). A and B refer to the different trial arms of each study. Details of which are provided in Table 2

Hypoxic exposure resulted in a non-significant increase in absolute carbohydrate oxidation rates during exercise matched for *absolute* intensities, compared with normoxia (mean difference = 0.21 g·min^− 1^, 95% CI = − 0.11 to 0.53; *n* = 6, *p* = 0.19; Fig. 2). The degree of heterogeneity was found to be high between studies (*I*^2^ = 98.69%, Q = 380.53, τ^2^ = 0.15, d_f_ = 5). Sensitivity analysis revealed that the removal of one comparison by Braun et al. [7] increased the mean difference to 0.32 g·min^− 1^ (95% CI: 0.18 to 0.47; *p* = 0.01). Inspection of the funnel plot and Egger’s regression intercept revealed little evidence of small study effects (intercept = 7.95, 95% CI: − 6.96 to 22.85; *p* = 0.21).

**Fig. 3:**
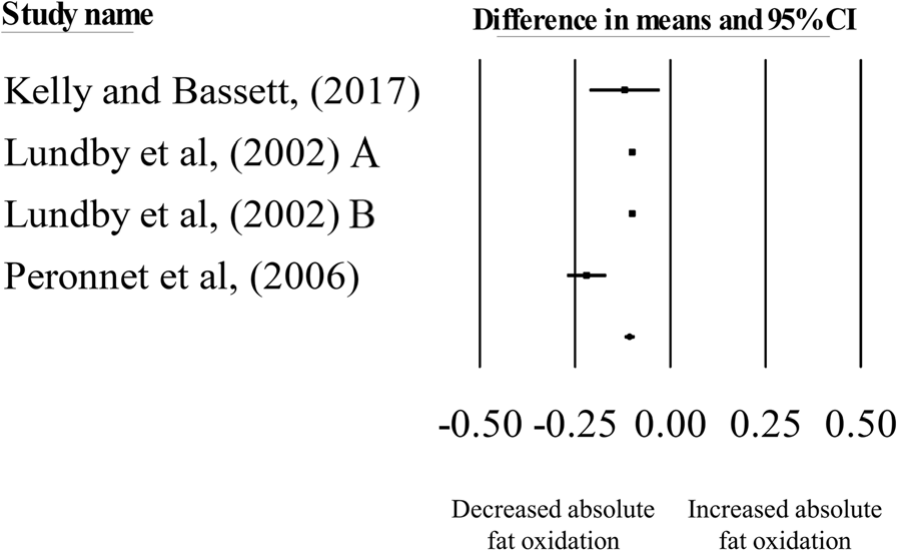
Forest plot of mean differences (means ±95% CI) for studies investigating the effects of hypoxia on absolute fat oxidation during exercise matched for absolute intensities. The size of the square represents the relative weight of the trial. CIs are represented by a horizontal line through their representative circles. The diamond quantifies the overall mean difference (means ±95% CI). A and B refer to the different trial arms of each study. Details of which are provided in Table 2

Hypoxic exposure resulted in a significant reduction in absolute fat oxidation during exercise matched for *absolute* intensity, compared with normoxia (mean difference: − 0.11 g·min^− 1^, 95% CI: − 0.12 to − 0.09; *n* = 4, *p* < 0.01; Fig. 3). The degree of heterogeneity was found to be high between studies (*I*^2^ = 85.85%, Q = 21.20, τ^2^ = 0.00009, d_f_ = 3). Sensitivity analysis revealed minor changes only, and these changes did not substantially alter the overall mean difference. Inspection of the funnel plot and Egger’s regression intercept revealed evidence of small study effects (intercept = − 2.64, 95% CI: − 9.59 to 4.31; *p* = 0.24).

### Risk of bias

**Fig. 4:**
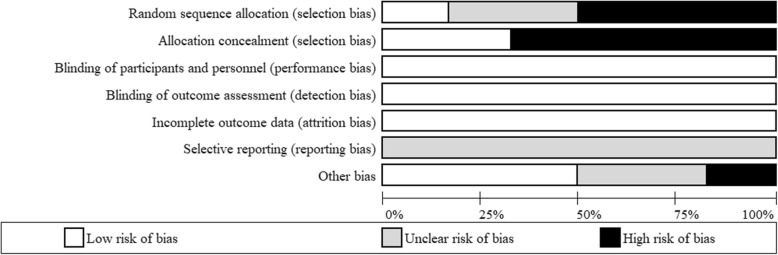
Assessment of risk of bias (Cochrane’s collaboration tool)

Since many of the studies were high altitude expeditions, certain biases were often unavoidable such as blinding of participants and personnel (Fig. 4). However, it was deemed that some of these biases could not affect the outcome variable and were therefore classified as low risk. In addition, all included studies were not clinically registered, therefore it is not possible to determine if all outcome variables were reported, therefore selective reporting bias was listed as unclear.

## Discussion

In response to Young et al. [1], the purpose of this manuscript was to examine the effects of hypoxic exposure on substrate oxidation during exercise matched for *absolute* intensities. Findings from this meta-analysis support those reported by Young et al. [1] but highlight some interesting discussion points. We observed an increased relative carbohydrate contribution to energy provision during exercise matched for *absolute* intensities in hypoxia compared with normoxia. A concurrent reduction in the relative contribution of fat to energy provision during exercise matched for *absolute* intensities was also observed. This effect was not moderated by any of the experimental characteristics included in this analysis, likely due to the dominant effect of an increased exercise stimulus. Notably, this contrasts our previously reported data demonstrating no difference in the relative contribution of carbohydrate or fat to energy provision during exercise matched for *relative* intensities in hypoxia compared with normoxia [2].

A greater RER and an increase in relative (but not absolute) carbohydrate oxidation were observed in hypoxia when exercise was matched for *absolute* intensities. These findings are likely due to the reduced $\dot{V}$O_2max_ experienced in hypoxia [13], and subsequent increase in relative exercise intensity for a given workload [10]. The physiological mechanisms associated with these changes in substrate oxidation are likely explained as per the normoxic response to increased exercise intensity, as detailed previously [2]. Interestingly, these data contrast with data reported by Young et al. [5] who observed no significant change in absolute whole body carbohydrate oxidation during exercise matched for *absolute* intensities in acute hypoxia (terrestrial altitude ~ 4300 m) compared with normoxia with supplementation of a glucose and fructose beverage. These findings are surprising given the aforementioned effect of an increased relative exercise intensity on substrate oxidation and demonstrate the need for further research to elucidate these responses.

At the time of analysis, the small number of studies investigating exogenous/endogenous carbohydrate oxidation meant these data were not appropriate for inclusion in a meta-analysis. Young et al. [1] summarised that exogenous carbohydrate oxidation may be suppressed during exercise matched for *absolute* intensities in acute hypoxia compared with normoxia, however due to the paucity of research in this area, this response remains to be established. However, recent data from O’Hara et al. [6] investigating substrate oxidation responses in females during exercise matched for *relative* intensities in hypoxia and normoxia may somewhat support this suppression of exogenous carbohydrate oxidation. The efficacy of carbohydrate supplementation to improve exercise performance is likely determined by our ability to oxidise exogenous carbohydrate sources. Thus, future research is required to determine this response and establish the performance effect of carbohydrate supplementation in hypoxia.

## Conclusions

Previously unpublished data from our recent meta-analysis confirms evidence provided by Young et al. [1], in demonstrating an increased relative contribution of carbohydrate oxidation to energy provision during exercise matched for *absolute* intensities in hypoxia compared with normoxia. These data now provide a comparable dataset (*relative* vs. *absolute* intensities) for use by researchers and practitioners in the design of nutritional interventions for relevant populations.

## Supplementary information

**Additional file 1.** Summary of moderator variables from the meta-regression model for RER in response to hypoxic exposure during exercise matched for absolute intensities.

**Additional file 2.** Forest plot of mean differences (means ±95% CI) for studies investigating the effects of hypoxia on relative carbohydrate oxidation during exercise matched for absolute intensities. The size of the square represents the relative weight of the trial. CIs are represented by a horizontal line through their representative circles. The diamond quantifies the overall mean difference (means ±95% CI). A and B refer to the different trial arms of each study. Details of which are provided in Table 2.

**Additional file 3.** Forest plot of mean differences (means ±95% CI) for studies investigating the effects of hypoxia on relative fat oxidation during exercise matched for absolute intensities. The size of the square represents the relative weight of the trial. CIs are represented by a horizontal line through their representative circles. The diamond quantifies the overall mean difference (means ±95% CI). A and B refer to the different trial arms of each study. Details of which are provided in Table 2.

## Data Availability

The data analysed and generated in this study are included in this published article and the associated additional files.

## Abbreviations

AH: Acute hypoxia
CH: Chronic hypoxia
CHO: Carbohydrate
CI: Confidence interval
HH: Hypobaric hypoxia
MD: Mean difference
NH: Normobaric hypoxia
NM: Not measured
RER: Respiratory exchange ratio
SD: Standard deviation
SL: Sea level
TA: Terrestrial altitude
