# Supplementary material for: Response: Commentary on the effects of hypoxia on energy substrate use during exercise
Source: J Int Soc Sports Nutr. 2019 Dec 19;16:61. doi: 10.1186/s12970-019-0330-7 (PMC6924012; doi:10.1186/s12970-019-0330-7)
Supplement: Supplementary file 1 — Additional file 1. Summary of moderator variables from the meta-regression model for RER in response to hypoxic exposure during exercise matched for absolute intensities. [file 12970_2019_330_MOESM1_ESM.docx]

| **Moderator variable**  **RER (relative) (n= 7)** | ***p* value** | **Comparison** |
| --- | --- | --- |
| Pre-existing nutritional state | 0.34 | Fasted (n = 2, MD 0.08, 95% CI -0.04 to 0.20) Fed (n = 5, MD 0.02, 95% CI 0.00 to 0.05) |
| Carbohydrate supplementation during exercise | 0.27 | Yes (n = 3, MD 0.01, 95% CI -0.02 to 0.05) No (n = 4, MD 0.06, 95% 0.03 to 0.09) |
| Exercise mode | N/A | All studies used cycling protocols |
| Duration of hypoxic exposure | 0.15 | Acute (n = 4, MD 0.06, 95% CI 0.03 to 0.10) Chronic (n = 9, MD 0.00, 95% CI -0.02 to 0.04) |
| Type of hypoxia | 0.38 | Simulated normobaric hypoxia (n = 3, MD 0.07, 95% CI 0.00 to 0.13) Simulated hypobaric hypoxia (n = 1, MD 0.05, 95% CI 0.04 to 0.06) Terrestrial altitude (n = 3, MD 0.01, 95% CI -0.02 to 0.04) |
| Percentage male | 0.21 | Meta-regression percentage male vs. MD (slope 0.0007, 95% CI -0.0006 to 0.0021) |
| Exercise intensity | 0.72 | Meta-regression of exercise intensity vs. MD (slope -0.0008, 95% CI -0.0061 to 0.0045) |
| Exercise duration | 0.55 | Meta-regression of exercise duration vs. MD (slope -0.0005, 95% CI -0.0025 to 0.0015) |
| Altitude height | 0.98 | Meta-regression of altitude height vs. ES (slope -0.00, 95% CI -0.0001 to 0.0001) |
