# Supplementary figures and images for: Response: Commentary on the effects of hypoxia on energy substrate use during exercise
Source: J Int Soc Sports Nutr. 2019 Dec 19;16:61. doi: 10.1186/s12970-019-0330-7 (PMC6924012; doi:10.1186/s12970-019-0330-7)

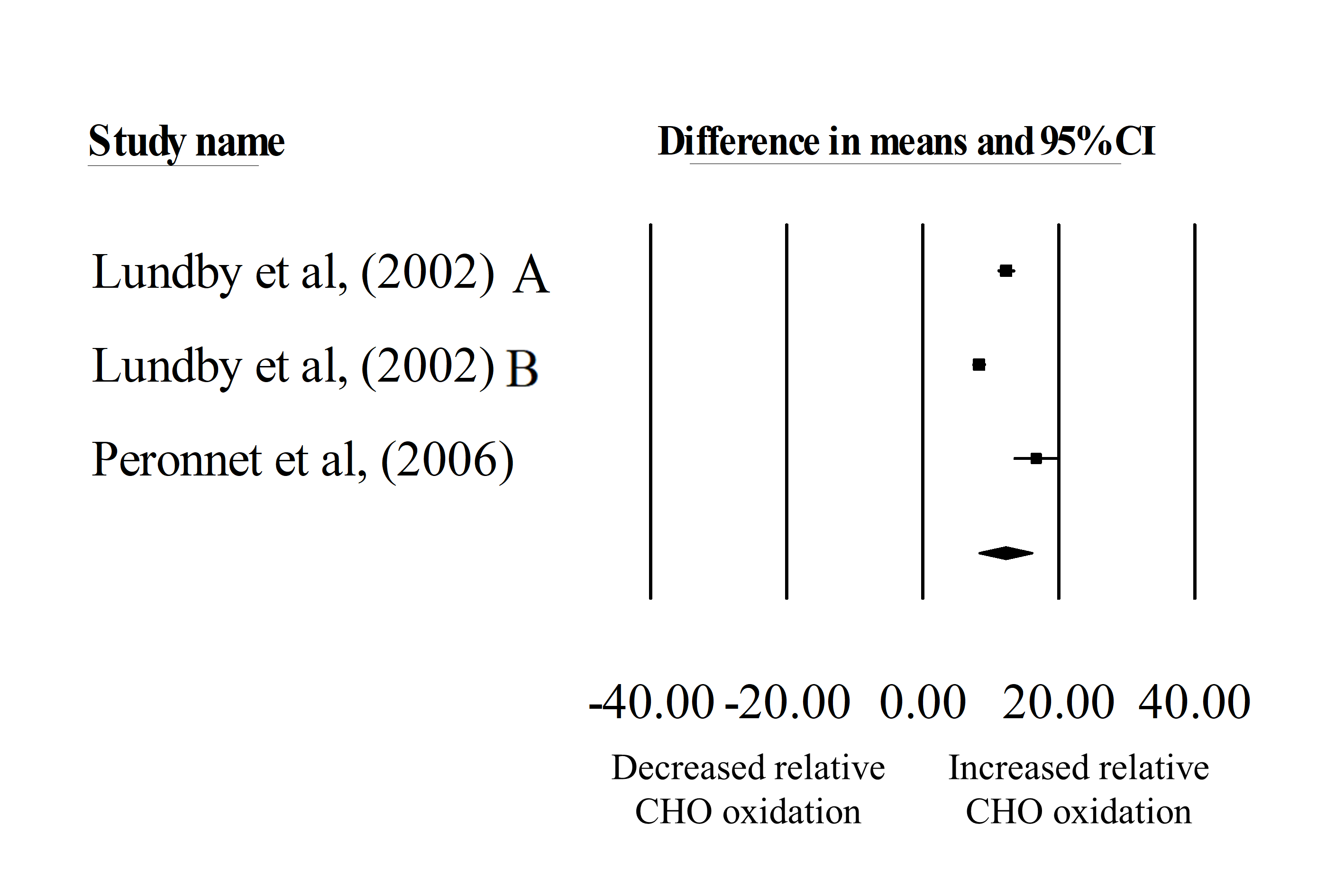

Supplement: Supplementary file 2 — Additional file 2. Forest plot of mean differences (means ±95% CI) for studies investigating the effects of hypoxia on relative carbohydrate oxidation during exercise matched for absolute intensities. The size of the square represents the relative weight of the trial. CIs are represented by a horizontal line through their representative circles. The diamond quantifies the overall mean difference (means ±95% CI). A and B refer to the different trial arms of each study. Details of which are provided in Table 2. [file 12970_2019_330_MOESM2_ESM.tif]

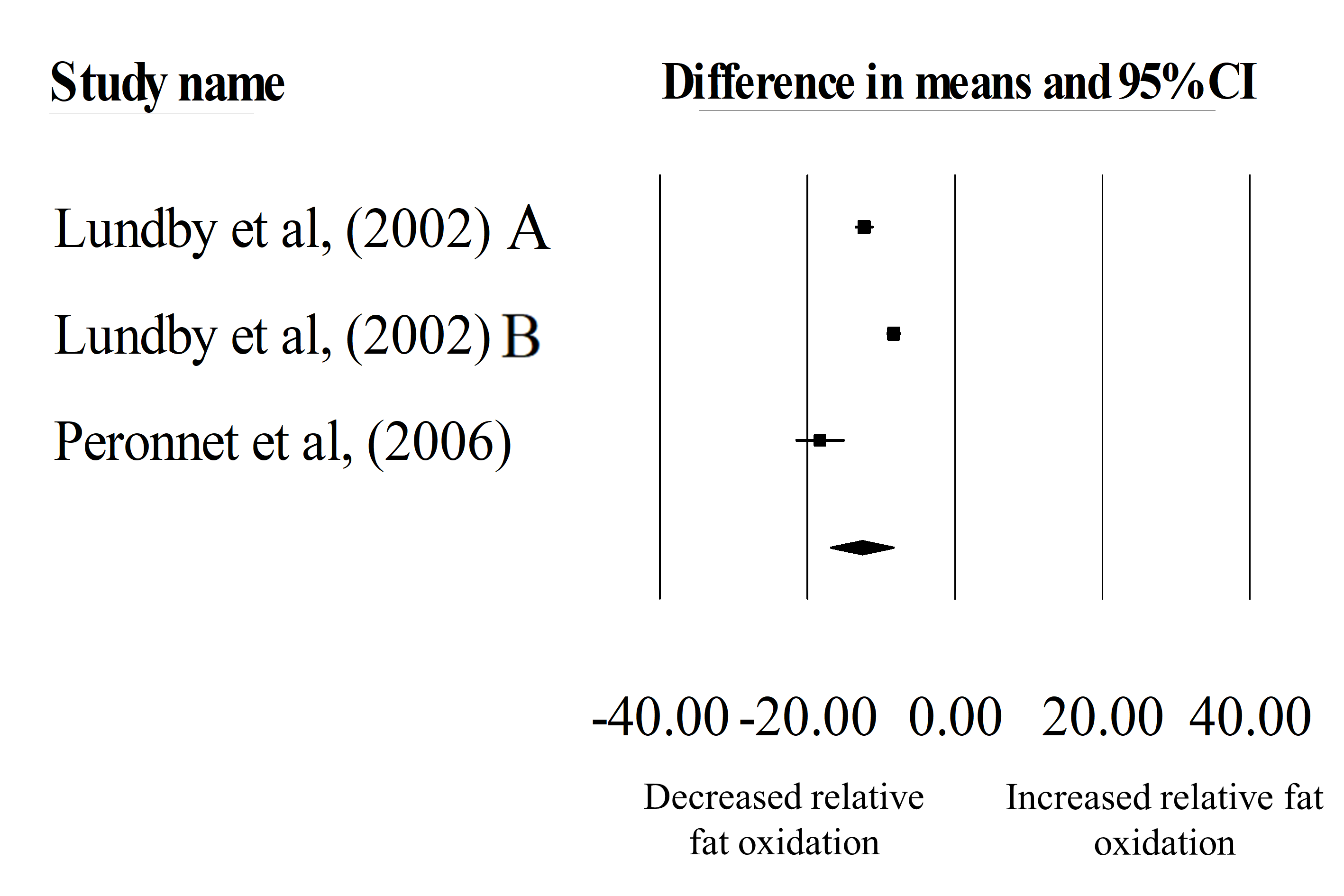

Supplement: Supplementary file 3 — Additional file 3. Forest plot of mean differences (means ±95% CI) for studies investigating the effects of hypoxia on relative fat oxidation during exercise matched for absolute intensities. The size of the square represents the relative weight of the trial. CIs are represented by a horizontal line through their representative circles. The diamond quantifies the overall mean difference (means ±95% CI). A and B refer to the different trial arms of each study. Details of which are provided in Table 2. [file 12970_2019_330_MOESM3_ESM.tif]
